# “I DON’T KNOW WHO GETS MORE OUT OF IT”: OLDER ADULTS’ EXPERIENCES WITH THE ONLINE INTERGENERATIONAL TUTORING PROGRAM

**DOI:** 10.1093/geroni/igad104.1130

**Published:** 2023-12-21

**Authors:** Edward Miller, Jessica Hoffman, James Hermelbracht, Jeffrey Burr, Jan Mutchler

**Affiliations:** University of Massachusetts Boston, Boston, Massachusetts, United States; Northeastern University, Boston, Massachusetts, United States; University of Massachusetts Boston, Boston, Massachusetts, United States; University of Massachusetts Boston, Boston, Massachusetts, United States; University of Massachusetts Boston, Boston, Massachusetts, United States

## Abstract

Due to pandemic-related academic and social stressors experienced by children and social isolation and loneliness experienced by older adults, a team of school psychologists and gerontologists collaborated with older adults to develop the Online Intergenerational Tutoring Program. Conceived in fall 2020, the program is now in its third year of implementation. The program addresses a service delivery gap in schools because older adult volunteers expand schools’ capacity to implement reading instruction with students in need of support. Older adults share their time and talents with young children and children benefit from the care, attention, and instruction from older adults. Tutors meet with students after school 3-4 times per week over a 6–8-week period over Zoom; each of the 24 sessions last 30 minutes. Evaluation data show the program is socially valid, tutors deliver instruction with accuracy, students have high engagement, and there are literacy gains among students.

